# Highly improved cloning efficiency for plasmid-based CRISPR knock-in in *C. elegans*

**DOI:** 10.17912/micropub.biology.000499

**Published:** 2021-11-19

**Authors:** Ella DeMott, Daniel J Dickinson, Ryan Doonan

**Affiliations:** 1 Glow Worms Stream, Freshman Research Initiative, Texas Institute for Discovery Education in Science, College of Natural Sciences, The University of Texas at Austin, Austin TX, USA; 2 Department of Molecular Biosciences, The University of Texas at Austin, Austin TX, USA

## Abstract

Plasmid-based CRISPR knock-in is a streamlined, scalable, and versatile approach for generating fluorescent protein tags in *C. elegans *(Dickinson *et al*. 2015; Schwartz and Jorgensen 2016). However, compared to more recent protocols that utilize commercially available Cas9/RNP products and linear DNA repair templates (Dokshin *et al*. 2018; Ghanta and Mello 2020), the cloning required for plasmid-based protocols has been cited as a drawback of this knock-in approach. Using thorough quantitative assessment, we have found that cloning efficiency can reproducibly reach 90% for the plasmids of the self-excising cassette (SEC) selection method, essentially resolving cloning as a burden for plasmid-based CRISPR knock-in.

**Figure 1. Highly improved cloning efficiency for plasmid-based CRISPR knock-in:**
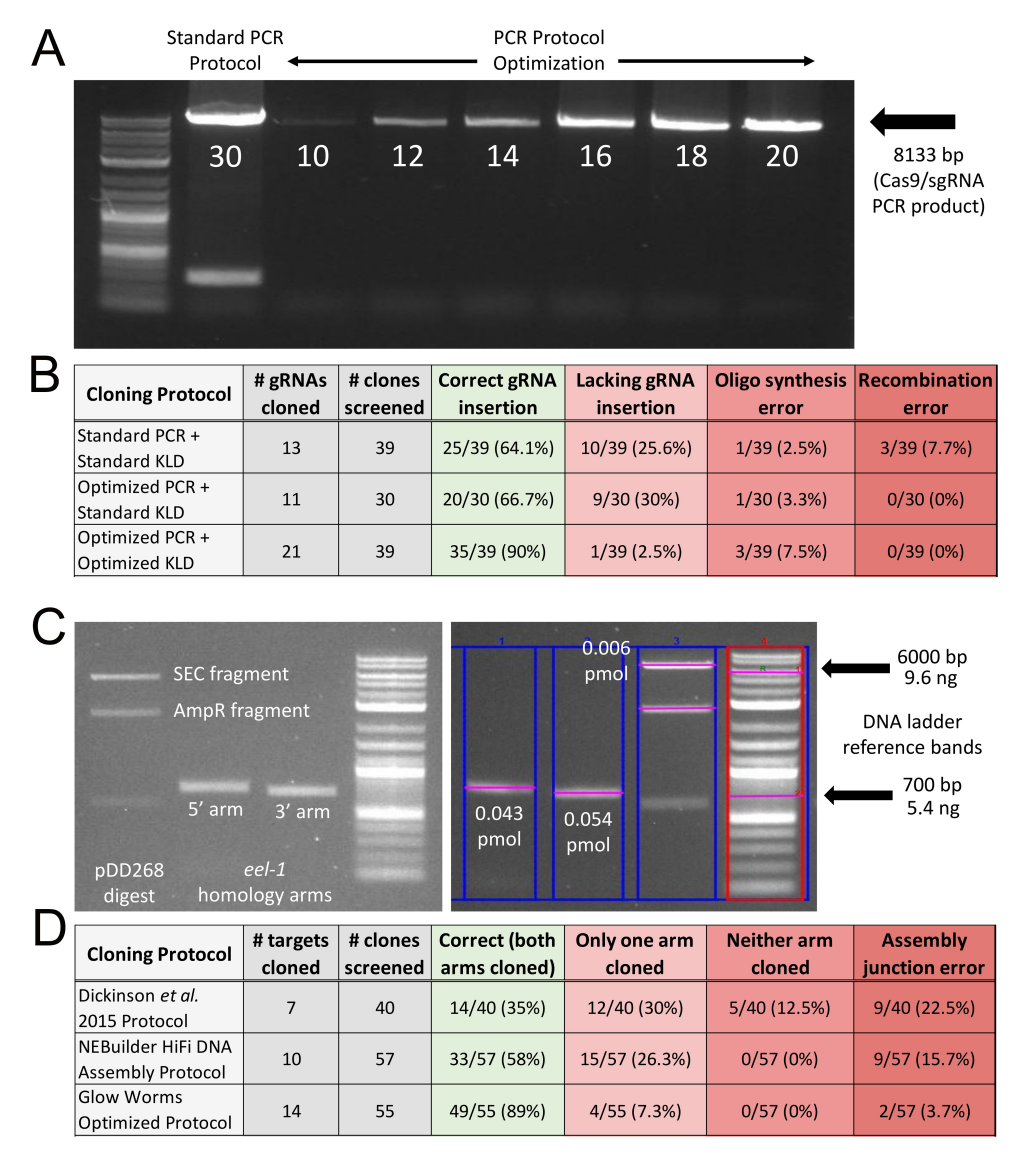
(A) Lane 1: NEB® DNA ladder standard (N3200S); Lane 2: A standard NEB® Q5 PCR protocol of 30 cycles results in too much DNA based on light smearing and an additional unwanted PCR product of ~300bp that reproducibly appears with 30 cycles; Lanes 3-8: Optimizing PCR cycle number for pDD162 amplification. Based on this data, we chose 15 cycles as the optimal number for all subsequent amplifications of pDD162. (B) Too much PCR product and insufficient DpnI digestion results in ~35% of KLD ligation clones being incorrect Cas9/sgRNA plasmids. In contrast, optimizing both the PCR and KLD ligation reactions results in 90% of clones having a correct gRNA insertion. (C) Case study of DNA yields for the vector backbone and homology arms used for construction of an FP/SEC repair plasmid, in this case for the gene *eel-1*. Left panel: post clean-up electrophoresis of a pDD268 SpeI/ClaI digestion and *eel-1* homology arm PCRs. Right panel: Another gel electrophoresis of the same pDD268 digestion and *eel-1* homology arms, this time with DNA quantification using Bio-Rad Image Lab 6.0 software to measure the intensity of ethidium bromide staining relative to an NEB® DNA ladder standard (N3200S). The 6000bp band of the ladder was used to determine the number of ng of SEC fragment and the 700bp band for the number of ng of each homology arm. Number of pmol was calculated as described in Methods. (D) Thorough quantification of DNA and subsequent optimization of the amount of DNA in the Gibson assembly reaction dramatically improves cloning efficiency for the FP/SEC plasmid, reproducibly reaching 89%. The “Glow Worms Optimized Protocol” is detailed in Methods.

## Description

The Glow Worms research stream is a course-based undergraduate research education (CURE) laboratory that focuses on making fluorescent protein knock-in strains at genetic loci of interest to the research community. To facilitate making strains, we aimed to determine whether the self-excising cassette (SEC) selection method for CRISPR knock-in could be improved. Indeed, we recently reported that using high-quality DNA and reducing Cas9/sgRNA concentration facilitates recovery of homology directed repair (HDR) knock-ins, requiring microinjection of only 10 P0 worms (Huang *et al.* 2021). Here, we show that cloning of SEC-based CRISPR plasmids can also be highly efficient and reproducible when optimized based on quantitative considerations.

Construction of the Cas9/sgRNA plasmid requires inserting a unique gRNA sequence into plasmid pDD162 (Dickinson *et al.* 2013) or one of its derivatives (Ward 2015; Aljohani *et al.* 2020) via site-directed mutagenesis. This involves a PCR step, followed by a KLD ligation step. Using a standard NEB® Q5 PCR protocol of 30 cycles (M0491) and a standard NEB® KLD reaction protocol (M0554), we found that 64.1% of clones included a correct gRNA insertion (Fig. 1A, B). Incorrect clones could be categorized into one of three groups: (1) those lacking the gRNA insertion (i.e. the pDD162 PCR template was transformed instead); (2) those lacking a base or two at the 5’ end of the gRNA (i.e. presumably the result of oligos in the primer pool with incomplete synthesis errors); or (3) those where a reproducible recombination of pDD162 had occurred (Fig. 1B). Based on the types of incorrect clones observed, we hypothesized that we were both using too much DNA in the KLD reaction and that the 5 minute room temperature incubation was insufficient for DpnI to digest the methylated pDD162 template prior to transformation. Indeed, limiting the PCR to 15 cycles and inclusion of a 20 minute 37°C incubation step in the KLD ligation protocol resulted in 90% of clones being correct Cas9/sgRNA plasmids (Fig. 1A, B). This is highly cost effective, as we now routinely transform with as little as 10 µL of competent cells and only need to screen one clone.

Construction of the FP/SEC plasmid requires insertion of two PCR-amplified homology arms into a pre-digested plasmid via Gibson assembly (Dickinson *et al.* 2015). Using the published protocol, which specifies a 4:1 volume ratio of purified homology arms to purified vector DNA (Dickinson *et al.* 2015), we found that only 35% of clones were correct assemblies, and the percentage of correct clones was highly variable among unique assembly reactions (Fig. 1D). Indeed, sometimes we would need to screen as many as 10 clones to identify a correctly assembled plasmid. This low efficiency was most likely due to both student inexperience with cloning and inconsistencies in the amount of DNA being used in the assemblies. Thus, we decided to take a more quantitative approach, following the recommendations of the NEBuilder® HiFi DNA Assembly protocol for a four fragment assembly, which specifies a 1:1 ratio of homology arms and vector DNA at 0.05 pmol each for all fragments. We quantified the DNA using ethidium bromide staining, an NEB® DNA ladder standard (N3200S), and Bio-Rad Image Lab 6.0 software (Fig. 1C). This definitely resulted in improved efficiency, yielding 58% of clones with correct assemblies (Fig. 1D). Nevertheless, the frequency of assembly errors remained high (42%), so we wondered whether we were again using too much DNA. We reduced the total amount of DNA 16-fold and modified the protocol to a 2:1 ratio of homology arms to vector DNA, which we based on our typical yield of homology arm and vector DNA following spin column clean-up, as well as limiting the total reaction volume to 10 µL (see Methods). Remarkably, this resulted in a highly reproducible success rate, with an average of 89% of clones being correct for 14 unique assemblies representing 14 different gene targets (Fig 1D). Thus, we now routinely need to screen just one or two clones per assembly, and oftentimes 100% of the clones are correct when screening three or more.

Overall, in combination with our previous report of highly improved knock-in efficiency via the SEC selection method (Huang *et al.* 2021), our new finding that SEC-based plasmids can be reproducibly cloned at 90% efficiency has essentially resolved the two major drawbacks of plasmid-based CRISPR knock-in. As such, plasmid-based approaches are equally attractive relative to recent approaches utilizing commercially available Cas9/RNP products and linear DNA repair templates that do not require any cloning (Dokshin *et al.* 2018; Ghanta and Mello 2020). As an undergraduate CURE, plasmid-based approaches are more cost effective, much more scalable to a large number of students, an excellent educational opportunity for molecular cloning, and plasmids are a permanent archive of sequence-verified clones that can be relatively easily repurposed for alternative tags such as other fluorescent markers or auxin-inducible degron (AID) (Ashley *et al.* 2021). We anticipate that our improvements will make plasmid-based CRISPR knock-in accessible for a wider range of researchers.

## Methods

*List of genes selected for cloning of gRNAs and homology arms in this study (45)*

· *amph-1, asp-3, asp-4, attf-2, ccdc-47, cdc-37, cey-1, cey-4, daf-18, dpy-10, edc-3, eel-1, egl-1, egl-19, fust-1, gyf-1, hsp-4, htz-1, hum-6, irg-1, laf-1, lin-5, mcm-5, mpk-1, mtl-1, nhr-6, nhr-8, npp-9, npp-10, pek-1, pqn-59, ptl-1, rab-7, rack-1, ruvb-1, sod-3, spt-16, F08G12.1, F13E6.1, H34C03.2, T13C2.6, T28D6.6, W08E12.7, Y38H6C.16, ZK1058.9*

*Cas9/sgRNA plasmid PCR*

· Standard Q5 PCR protocol: 5 ng of pDD162 template, 61°C annealing, 30 cycles

· Optimized Q5 PCR protocol: 5 ng of pDD162 template, 65°C annealing, 15 cycles

*Cas9/sgRNA plasmid KLD ligation*

· KLD reaction: 1 µL of unpurified PCR product + 8.5 µL of KLD MM + 0.5 µL of KLD enzyme mix

· Standard KLD protocol: 5 min incubation at room temperature

· Optimized KLD protocol: 10 min incubation at room temperature, then 20 min incubation at 37°C

*Purification of FP/SEC plasmid DNA fragments*

Digested vector (e.g. pDD268 for mNeonGreen tags) and homology arm PCR products were purified using NEB® Monarch miniprep columns as follows:

(1) Dilute the volume of the digest and the PCRs to 40 µL each using ultrapure H_2_O.

(2) Add 200 µL of Qiagen buffer PB to each diluted reaction for a total of 240 µL.

(3) Purify the digestion and each homology arm separately using Monarch miniprep columns.

(4) Wash the columns with 400 µL of Monarch Wash Buffer 2.

(5) Elute the columns with 30 µL of Monarch Elution Buffer.

*Quantification of FP/SEC plasmid DNA fragments*

Purified DNA was quantified as follows:

(1) Run 1 µL of each purified DNA elution on the same 1% agarose gel containing ethidium bromide along with 2 µL of 0.02 µg/µL of DNA ladder standard.

(2) DNA band intensity was quantified relative to ladder using a Bio-Rad EZ Gel Doc system and Bio-Rad Image Lab 6.0 software.

(3) For each DNA fragment: [ng DNA x 1000]/[bp of DNA x 650 daltons] = # pmol/µL

*Protocol variants for Gibson assembly of FP/SEC plasmid DNA fragments*

· Previously published protocol (Dickinson *et al.* 2015): 4 µL of homology arms + 1 µL of digested vector + 5 µL of NEBuilder® HiFi DNA Assembly master mix

· NEBuilder® HiFi DNA Assembly protocol: 1:1 ratio of homology arms to digested vector, each fragment at 0.05 pmol in the reaction

· Glow Worms optimized protocol: 2:1 ratio of homology arms to digested vector, homology arm fragments at 0.004 pmol each and digested vector at 0.002 pmol each (i.e. 0.002 pmol of SEC fragment and 0.002 pmol of AmpR fragment).

*FP/SEC plasmid Gibson assembly reaction*

Gibson assembly reactions were incubated at 50°C for 1 hour and then either frozen at -20°C or used immediately for bacterial transformation.

## Reagents

**Table:** 

**Reagent**	**Supplier/Cat# or Recipe**
T4 polynucleotide kinase	NEB® M0201S
T4 DNA ligase	NEB® M0202S
T4 DNA ligase buffer	NEB® B0202S
DpnI	NEB® R0176S
“Homemade” KLD master mix (MM)	1 µL of T4 DNA ligase buffer + 9 µL of ultrapure H_2_O
“Homemade” KLD enzyme mix	16.7 µL kinase + 1 µL ligase + 8.3 µL DpnI
Monarch miniprep kit	NEB® T1010L
PB buffer	Qiagen 19066
DNA ladder standard	NEB® N3200S
HiFi DNA assembly master mix	NEBuilder® E2621L
